# Supplementary material for: Alternate oscillations of Martian hydrogen and oxygen upper atmospheres during a major dust storm
Source: Nat Commun. 2022 Nov 3;13:6609. doi: 10.1038/s41467-022-34224-6 (PMC9633821; doi:10.1038/s41467-022-34224-6)
Supplement: Supplementary file 1 — Supplementary Information [file 41467_2022_34224_MOESM1_ESM.pdf]

Supplementary Information for "Alternate oscillations of Martian hydrogen and oxygen upper atmospheres during a major dust storm"

Kei Masunaga*[1], Naoki Terada[2], Nao Yoshida[2], Yuki Nakamura[2,3], Takeshi Kuroda[2,4], Kazuo Yoshioka[5,6], Yudai Suzuki[6], Hiromu Nakagawa[2], Tomoki Kimura[7], Fuminori Tsuchiya[8], Go Murakami[1], Atsushi Yamazaki[1], and Ichiro Yoshikawa[5,6]

1. Institute of Space and Astronautical Science, Japan Aerospace Exploration Agency, Sagamihara, Japan
2. Department of Geophysics, Graduate School of Science, Tohoku University, Sendai, Japan
3. LATMOS, Sorbonne Université, Paris, France
4. Division for the Establishment of Frontier Sciences of Organization for Advanced Studies, Tohoku University, Sendai, Japan
5. Graduate School of Frontier Sciences, University of Tokyo, Kashiwa, Japan
6. Department of Earth and Planetary Science, Graduate School of Science, University of Tokyo, Tokyo, Japan
7. Faculty of Science, Tokyo University of Science, Tokyo, Japan
8. Planetary Plasma and Atmospheric Research Center, Graduate School of Science, Tohoku University, Sendai, Japan

Corresponding author: Kei Masunaga (masunaga.kei@jaxa.jp)

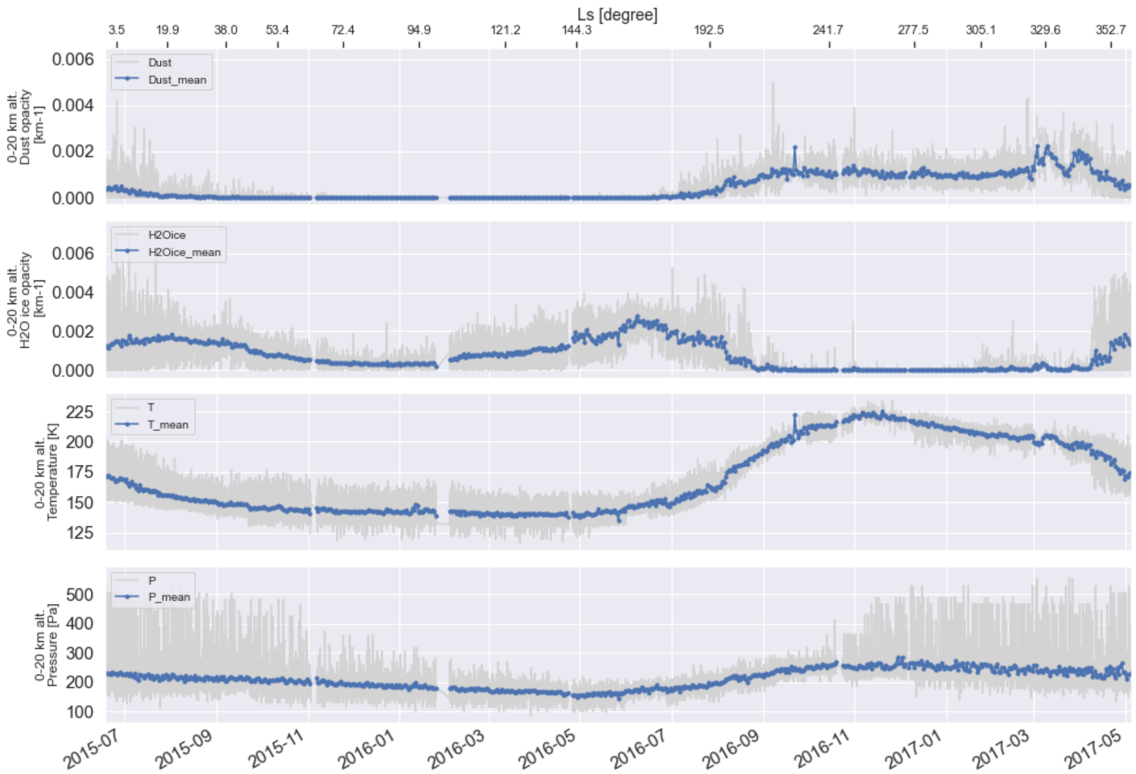

Supplementary Fig. 1. Mars Climate Sounder (MCS) measurements throughout Mars Year 33.

MCS measurements of dust opacity, H_2O ice opacity, air temperature, and air pressure observed at an altitude of 0–20 km at a local time of 11–13 h throughout Mars Year 33 (gray lines). The blue dots denote the average values of every Martian day, as shown in Figs. 1 and 2.

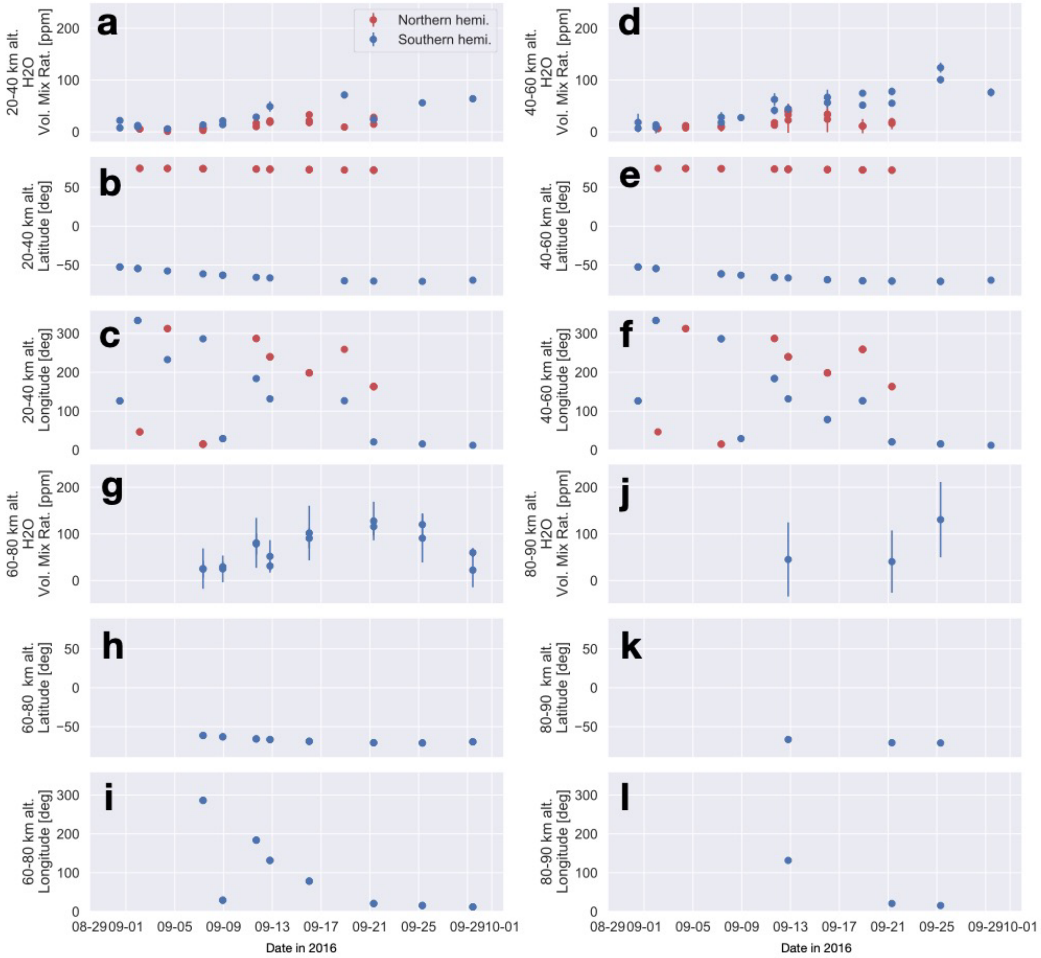

Supplementary Fig. 2. Spectroscopy for the Investigation of the Characteristics of the Atmosphere of Mars (SPICAM) measurements and its observation geometry.

SPICAM measurements of water vapor mixing ratio in four altitude ranges (20–40 km, 40–60 km, 60–80 km, and 80–90 km) and their observed latitude and longitude. The two colors (red and blue) denote measurements on the northern and southern hemispheres, respectively.

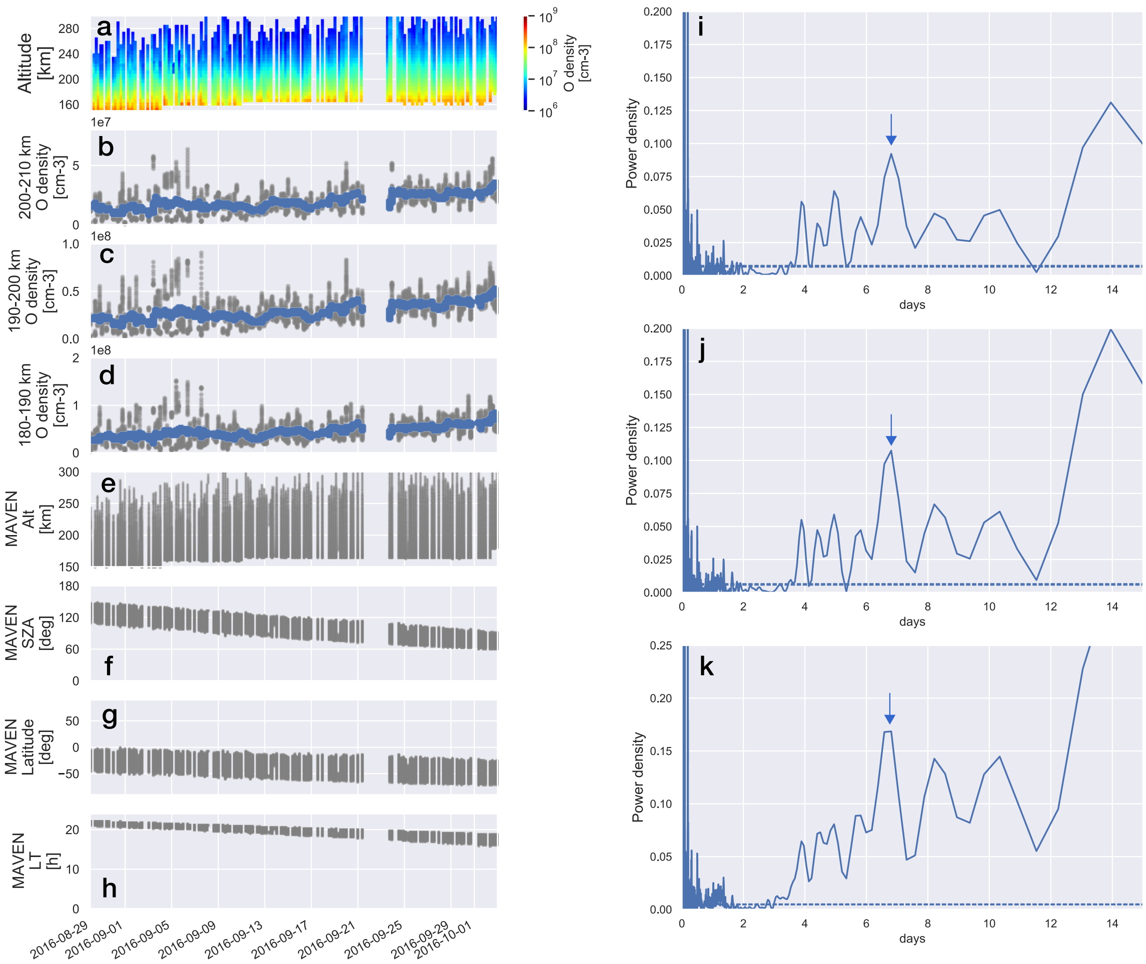

Supplementary Fig. 3. Neutral Gas and Ion Mass Spectrometer (NGIMS) measurements, its observation geometry, and periodogram result.

(**a-h**) NGIMS measurements of O number density in the 150–300 km altitude range and density variations at 200–210 km, 190–200 km, and 180–190 km altitudes are shown along with MAVEN geometric information. Grey dots represent original data, and blue dots are running average values using 1-day window. (**i-k**) Power spectra of O densities calculated using the periodogram method. Horizontal dashed lines show the 99 % confidence level and the blue arrows indicate the detected 6.8-day periodicity.

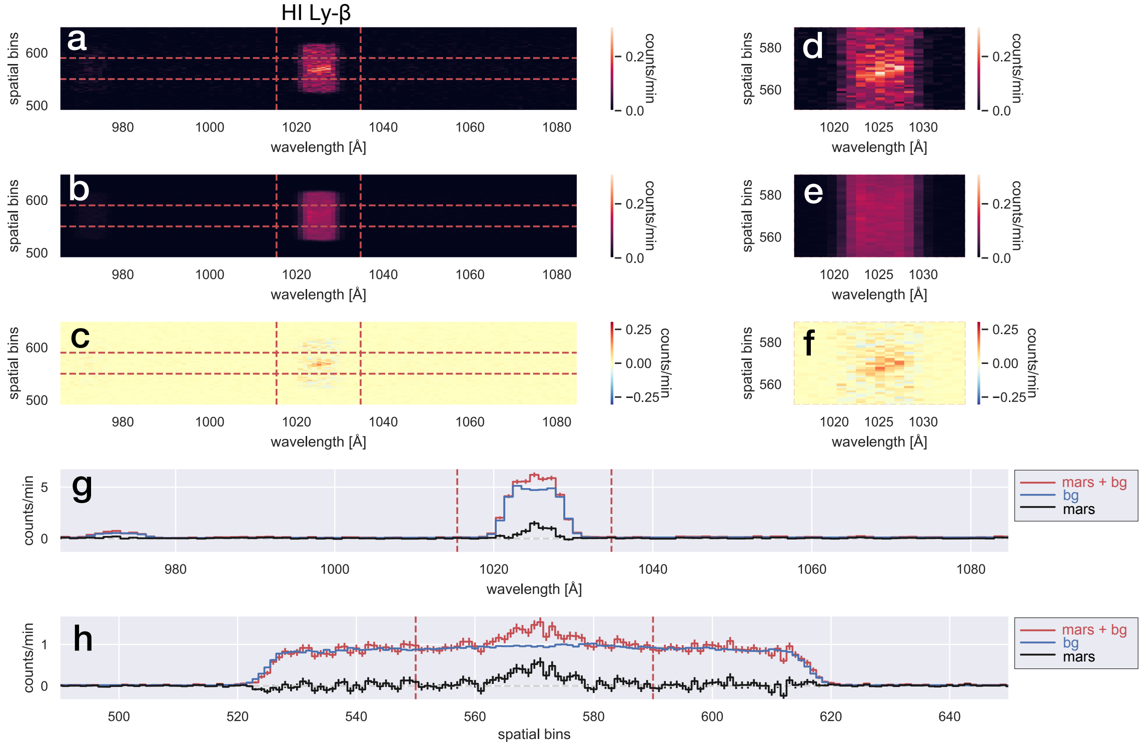

Supplementary Fig. 4. HI Ly- β airglow observed by Hisaki.

Average spectra of Ly-β for (**a**) on-Mars, (**b**) off-Mars (background), and (**c**) their residual observed on September 4, 2016. Zoom-in features are shown in panels (**d**), (**e**), and (**f**), respectively. Their spectral and spatial distributions are shown in panels (**g**) and (**h**).

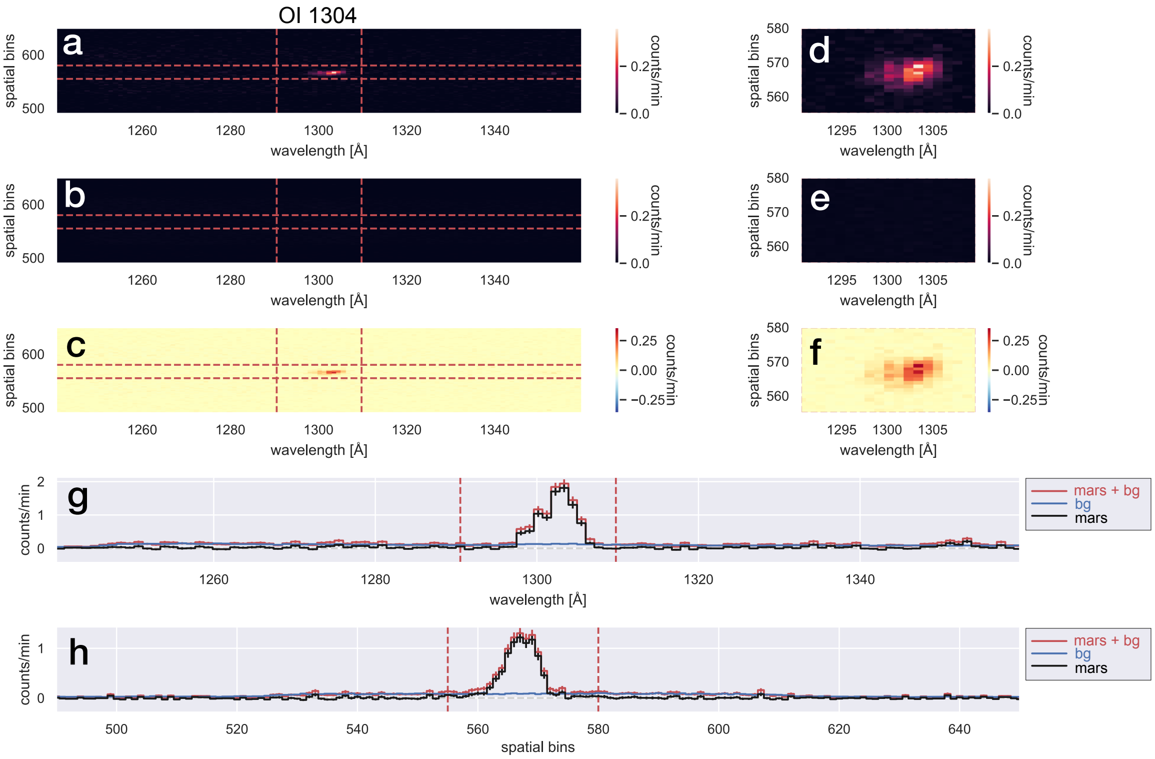

Supplementary Fig. 5. OI 1304 Å airglow observed by Hisaki.

Average spectra of OI 1304 Å observed on September 4, 2016 in the same format as Supplementary Fig. 4.

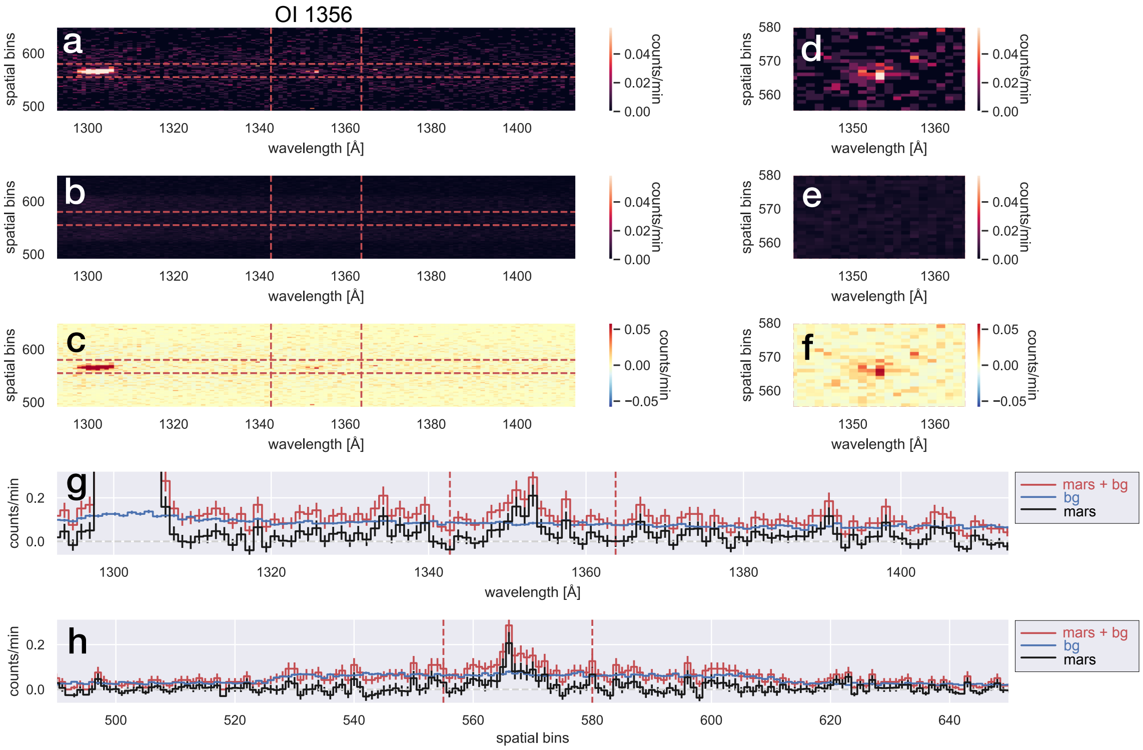

Supplementary Fig. 6. OI 1356 Å airglow observed by Hisaki.

Average spectra of OI 1356 Å observed on September 4, 2016 in the same format as Supplementary Fig. 4.

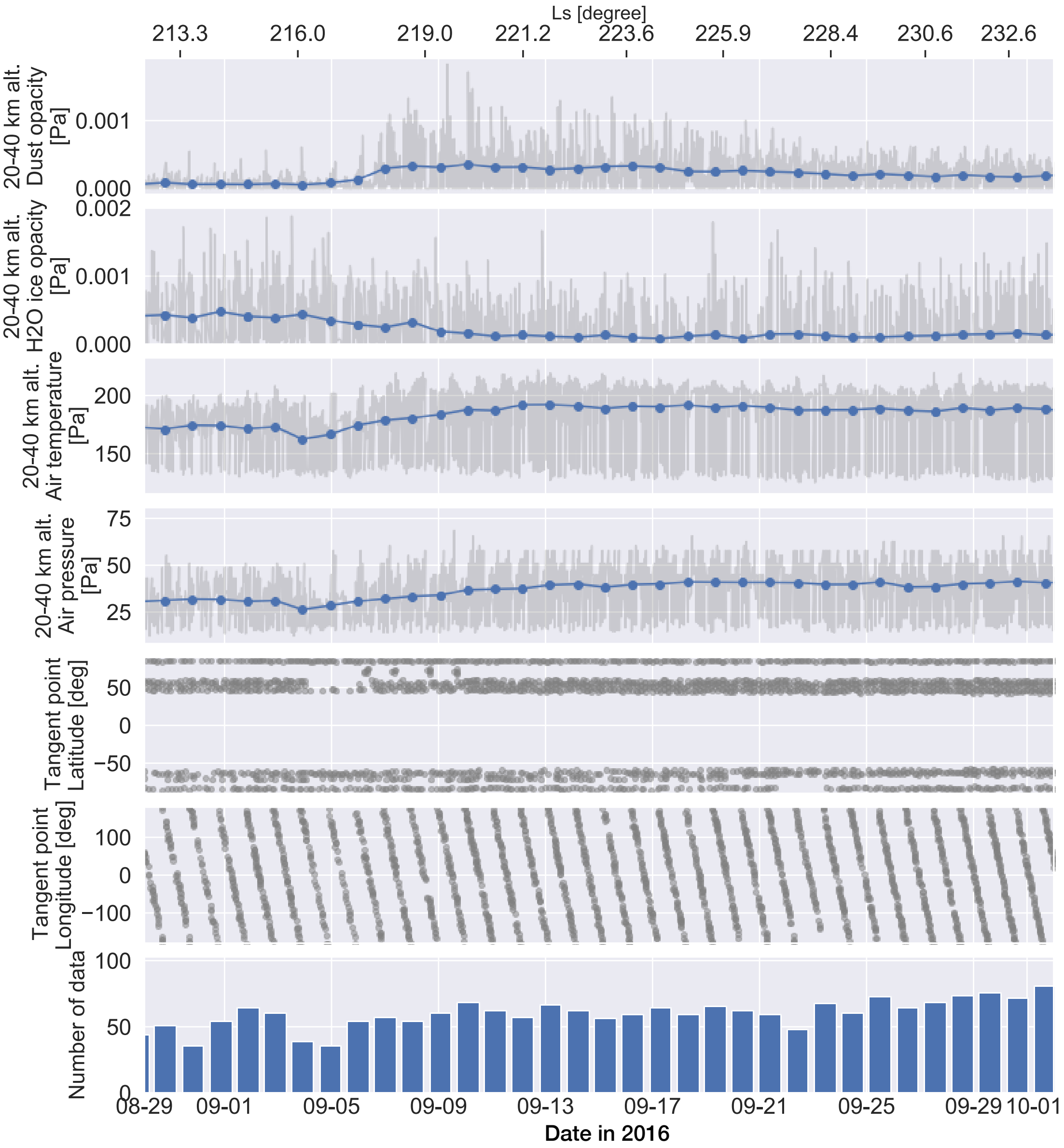

Supplementary Fig. 7. Mars Climate Sounder (MCS) measurements and its observation geometry.

The top four panels show MCS measurements of dust opacity, H_2O ice opacity, air temperature, and air pressure observed at an altitude of 20–40 km at a local time of 11–13 h (gray lines). The blue dots denote the average values of every Martian day, as shown in Figs. 1 and 2. Observation latitude, longitude, and the number of data points per Martian day are also shown.
