# Supplementary material for: Alternate oscillations of Martian hydrogen and oxygen upper atmospheres during a major dust storm
Source: Nat Commun. 2022 Nov 3;13:6609. doi: 10.1038/s41467-022-34224-6 (PMC9633821; doi:10.1038/s41467-022-34224-6)
Supplement: Supplementary file 2 — Description of Additional Supplementary Files [file 41467_2022_34224_MOESM2_ESM.docx]

**Description of Additional Supplementary Files**

File Name: Supplementary Data 1

Description: Timeseries of Ly-β, OI 1304 Å, and OI 1356 Å airglow observed by Hisaki telescope. The first column is the average time of each daily observation. The second and third columns are the deviation in seconds from the average time to indicate the first and last measurements of each daily observation. The fourth and ninth columns are the Ly-β, OI 1304 Å, and OI 1356 Å airglow brightness values and their standard deviations, respectively.
